# Association between early childhood exposure to malaria and children’s pre-school development: evidence from the Zambia early childhood development project

**DOI:** 10.1186/1475-2875-12-12

**Published:** 2013-01-08

**Authors:** Günther Fink, Analia Olgiati, Moonga Hawela, John M Miller, Beatrice Matafwali

**Affiliations:** 1 Harvard School of Public Health, 665 Huntington Avenue, Boston, MA 02115, USA; 2 Center for Population and Development Studies, Harvard University, 9 Bow Street, Cambridge, MA, 02139, USA; 3 National Malaria Control Centre (NMCC), Lusaka, Zambia; 4 PATH Malaria Control and Evaluation Partnership in Africa (MACEPA), Lusaka, Zambia; 5 University of Zambia, Lusaka, Zambia

**Keywords:** Malaria, Child development, Stunting, Underweight, Cognitive development

## Abstract

**Background:**

Despite major progress made over the past 10 years, malaria remains one of the primary causes of ill health in developing countries in general, and in sub-Saharan Africa in particular. Whilst a large literature has documented the frequency and severity of malaria infections for children under-five years, relatively little evidence is available regarding the impact of early childhood malaria exposure on subsequent child development.

**Methods:**

The objective of the study was to assess the associations between early childhood exposure to malaria and pre-school development. Child assessment data for 1,410 children in 70 clusters collected through the 2010 Zambian Early Childhood Development Project was linked with malaria parasite prevalence data from the 2006 Zambia Malaria Indicator Survey. Linear and logistic models were used to estimate the effect of early childhood exposure to malaria on anthropometric outcomes as well as on a range of cognitive and behavioural development measures.

**Results:**

No statistically significant associations were found between parasite exposure and children’s height and weight. Exposure to the malaria parasite was, however, associated with lower ability to cope with cognitive tasks administered by interviewers (z-score difference −1.11, 95% CI −2.43–0.20), as well as decreased overall socio-emotional development as assessed by parents (z-score difference −1.55, 95% CI −3.13–0.02). No associations were found between malaria exposure and receptive vocabulary or fine-motor skills.

**Conclusions:**

The results presented in this paper suggest potentially large developmental consequences of early childhood exposure to malaria. Continued efforts to lower the burden of malaria will not only reduce under-five mortality, but may also have positive returns in terms of the long-term well-being of exposed cohorts.

## Background

The importance of early child development on all dimensions of individual wellbeing in later stages of life has been well documented in developed countries. On average, developmental delays during early childhood are associated with poorer schooling outcomes, lower incomes, and substantially higher risks of ill health during adult life [1-4]. An estimated 200 million children in sub-Saharan Africa and Southeast Asia are assumed to remain below their developmental potential today [5]. While it is generally presumed that one of the principal drivers of delayed child development is in utero and early childhood health [5-7], causal evidence is still limited and particularly lacking in the context of infectious diseases in developing countries.

This paper examines the associations between exposure to malaria during the first two years of life and children’s subsequent development at age five. According to the latest World Health Organization (WHO) estimates, about half of the world’s population continues to be exposed to malaria today despite massive international efforts. Acute malaria is estimated to cause 225 million cases of ill health per year, resulting in over one million deaths per year, most of which occur in sub-Saharan Africa [8,9]. Malaria is particularly virulent among children, constituting one of the principal causes of child morbidity as well as mortality in sub-Saharan Africa [10]. Exposure to the malaria parasite not only results in bouts of high fevers among children, but also increases the risk of malnutrition and anaemia among children under five [11]. While several studies have highlighted the detrimental developmental impact of severe cerebral malaria during early childhood [12-18], relatively little evidence is available to date on the medium- to long-term effects of the exposure to malaria more generally [19]. Kihara *et al.*[20] reviewed the existing literature of the effects of *Plasmodium falciparum* on cognition, and found only one high-quality study which investigates the effect of asymptotic parasitaemia on cognition: in a study of school age children in Yemen, infected children were found to perform worse on fine motor skill tasks, but not to differ with respect to their cognitive scores two weeks after the initial infection [21]. Jukes *et al.*[22] followed up on a prophylaxis trial on children of ages 3–5 in the Gambia, and found no effect on cognition, but a positive effect of prophylaxis on school attainment.

To evaluate the associations between early childhood exposure to malaria and children’s physical, cognitive and socio-emotional development prior to school entry, cluster-level data on malaria parasite prevalence among children under the age of five from 2006 were combined with individual-level data from a detailed child development assessment conducted in 2010.

## Methods

**Figure 1:**
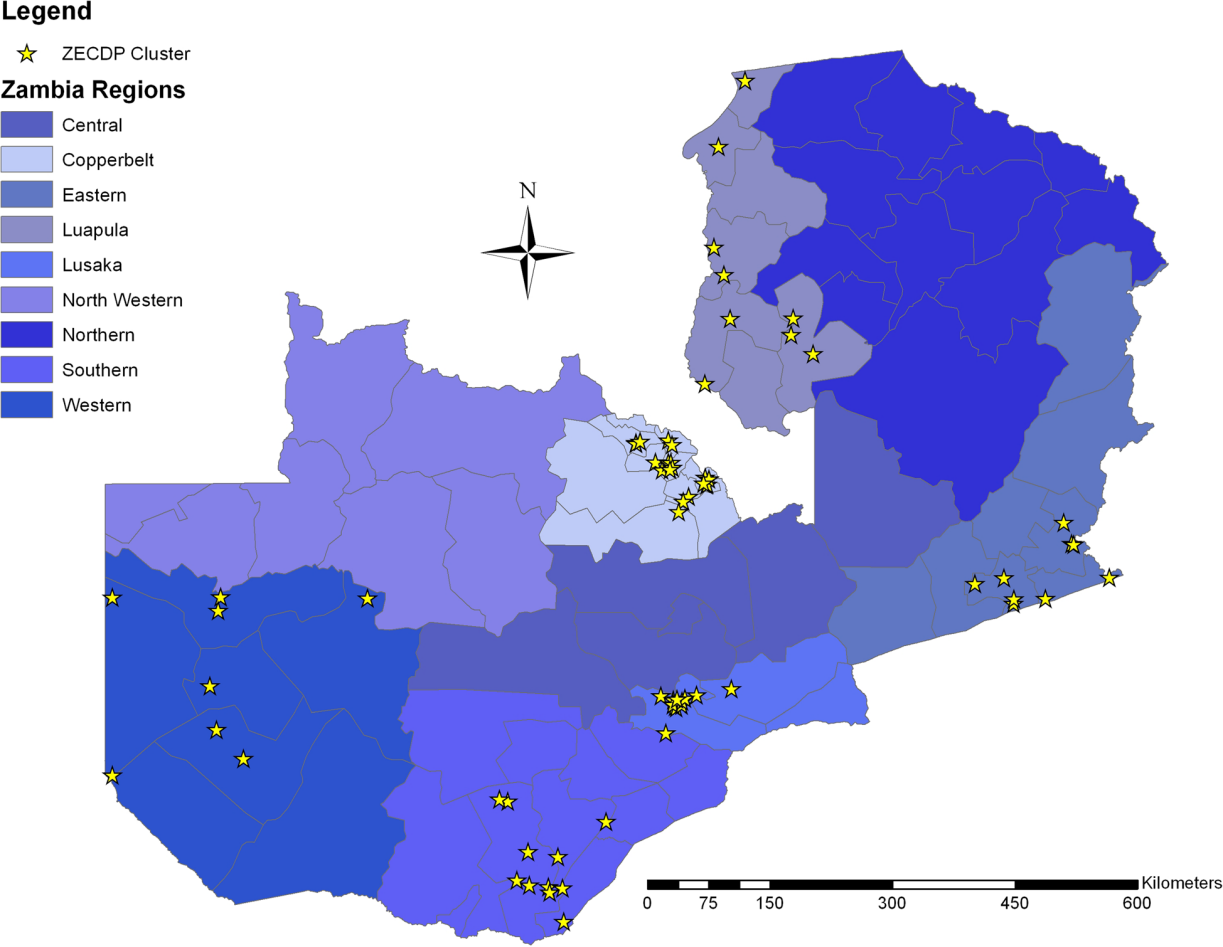
73 clusters sampled in the Zambia Early Childhood Development Project 2010 Survey.

The primary data on child development used for this project stem from the Zambia Early Childhood Development Project (ZECDP). Resulting from a joint effort between the Harvard Center on the Developing Child, the University of Zambia and UNICEF Zambia, the ZECDP was launched in 2009 with the objective to generate a comprehensive assessment of children’s development before entering school, using tools that are both internationally comparable and suitable for the sub-Saharan African context [23]. As part of this project, a first cohort of children born in 2004 was assessed with respect to their physical, cognitive and socio-emotional development between July and December 2010. The sampling frame used for the study was the 120 clusters surveyed in the 2006 Zambian Malaria Indicator Survey (MIS) [24]. In order to allow child assessments in one of the four most commonly spoken and curriculum designated languages, sampling was restricted to the six provinces where Nyanja, Bemba, Lozi, and Tonga are the predominantly spoken languages. This restriction resulted in a final ZECDP sample of 1,686 children in 73 clusters. As Figure 1 shows, sampling areas are more densely clustered in Lusaka and Copperbelt, but the sample covers a wide array of rural, urban as well as peri-urban areas.

As described in further detail in Fink *et al*[23], the ZECDP survey covered a wide range of child developmental outcomes. Four broad developmental domains were selected for the purpose of this study: physical development (anthropometrics), behavioural and socio-emotional development, receptive vocabulary (assessed through an adapted version of the Peabody Picture Vocabulary Test [25]) and general fine-motor skills as assessed by a series of tasks related to the child’s day-to-day activities as measures of school-readiness [26]. Language, fine motor skills and task orientation were assessed through a series of tests administered to children by study staff trained to conduct these assessments. Socio-emotional development was assessed through detailed questions about children’s behaviour answered by parents. Children’s height and weight was assessed using portable scales and height measures. Binary indicators for stunting (height for age 2 or more standard deviations below the reference median) and underweight (weight for age two or more standard deviations below the reference median) were calculated based on the AnthroPlus Software macro for Stata [27].

**Figure 2:**
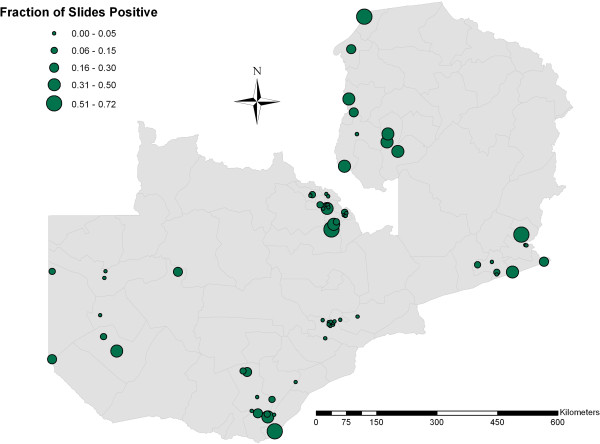
Blood slide positivity rates by clusters in the ZMIS 2006.

The key explanatory variable of interest was malaria exposure during early childhood. Instead of relying on parental self-reports, cluster-level parasitaemia data collected as part of the Zambia 2006 Malaria Indicator Survey (MIS) [24] were used to construct a village- or neighbourhood-specific measure of malaria exposure. As discussed in further detail in Riedel *et al.*[28], the MIS used rapid diagnostic tests and blood slides to test all children under the age of five in selected households for malaria parasites. On average, 17 malaria tests were collected in each cluster. As Figure 2 illustrates, parasitaemia (defined as the fraction of positive blood slides in the cluster) showed substantial variation across clusters and regions, with the highest rates on average observed for Eastern and Luapula region, and the lowest rates observed for Lusaka and the Copperbelt. While a large number of socioeconomic and behaviour factors may have affected malaria transmission, local geography and climate are generally considered the main determinants of malaria exposure [28,29].

Field work for the MIS was conducted between May and June 2006, which means that the children in the sample were between 18 and 29 months at the time of the MIS malaria testing. Even though children in the ZECDP sample cannot be linked to the MIS at the individual (child) level, the fact that generally all children born in 2004 in a given cluster were assessed in the ZECDP implies that the majority of children tested for malaria in the MIS were assessed as part of the ZECDP. Each study cluster corresponds to an enumeration area used in the Zambia 2000 census, and comprised about 100–250 households in 2000.

The MIS randomly selected about 10–15% households from each cluster for the survey, and then tested all children under-five for malaria. Since the ZECDP assessed more than 90% of children from the 2004 cohort living in each cluster, most children tested in the MIS can be presumed to be in the ZECDP sample. From the original ZECDP sample (N = 1,686), 221 children had moved since birth, and were excluded from the analysis. An additional three clusters (55 children) were excluded because no parasitaemia assessment was conducted in the MIS in their respective clusters, resulting in a final sample size of 1,410 children across 70 clusters with both child development and parasitaemia data.

Given that malaria-endemic areas are not only more rural on average, but presumably also poorer and exposed to a different set of risk factors, spatial correlations between malaria prevalence and child development cannot directly be interpreted as evidence of a causal impact of malaria on development. To at least partially address this spatial confounding concern, a large number of family as well and community characteristics were included in the empirical mode. At the time of the survey, Zambia was administratively divided into nine provinces and 72 districts – the 70 clusters in the sample are distributed across 32 of these 72 districts. All models estimated include district-fixed effects, which absorb all differences in the average prevalence levels across regions, so that the resulting empirical estimates purely explore within-district variations in malaria exposure. In order to limit the risk of confounding within districts, a large number of child-level controls, including the sex and age of the child in months, survival status of both parents, years of completed schooling of the parent or caretaker, as well as a range of household assets were included. As suggested by Filmer and Pritchett [30], principal component analysis was used to rank households with respect to their wealth, and divide households into wealth quintiles [31]. To further ensure that results are not driven by differences between poor high-prevalence rural clusters and low-prevalence urban areas, separate models were estimated for the rural sample only.

The statistical models estimated assess the empirical association between malaria exposure and several domains of child development: physical development, perceived behavioural and socio-emotional development, receptive vocabulary and fine-motor skills. As discussed above, all regressions control for district-fixed effects and child characteristics (sex and age), parent and household characteristics, as well as a set of cluster-level variables to address cluster-level confounding concerns. The multi-level model estimated can be described as follows

$$\begin{array}{l} y_{\mathrm{ic}}=\beta_{0}+\beta_{1}Malaria_{c}+X_{\mathrm{ic}}\gamma +\Phi_{c}\lambda +\left(\sum_{D=2}^{32}\delta_{D}I_{D}\right)+u_{\mathrm{ic},}\\ u_{\mathrm{ic}}=\varepsilon_{c}+\varepsilon_{\mathrm{ic}}\text{,} \end{array}$$

where *y*_*ic*_ is the developmental outcome of interest for child *i* in cluster *c, Malaria*_*c*_ is the 2006 malaria parasite prevalence in cluster *c* as assessed in the MIS, *X*_*ic*_ is a vector of child and household controls, Ф_*c*_ is a vector of cluster level controls, and *I*_*D*_ are the district fixed effects described above. The main outcome variables – the measures of child development – are observed at the child level, while each cluster or village was assumed to be characterized by a random intercept. More specifically, the error term *u*_*ic*_ in the empirical model was assumed to contain both a cluster-specific component *ε*_*c*_ and a truly random individual noise term *ε*_*ic*_. The random-intercept model estimated will yield unbiased estimates of the co-efficients of interest under the assumption that the cluster-specific intercepts are orthogonal to the other covariates included in the model [32].

In order to provide a better sense of the estimated magnitudes on malaria exposure, the estimated impact of reducing the exposure to malaria to zero was compared to two alternative changes in household structure: an increase in adult education, and an increase in household wealth. All estimates were implemented using the Stata© 11 software package.

## Results

Table 1 shows descriptive statistics for the entire sample. Forty-eight percent of children in the sample were male, with an average age of 75 months at the time of the assessment. Seven percent of children had lost their father, 1.9% had lost their mother, and 0.4% of children had lost both parents. While no detailed information on the cause of death or HIV positivity was collected, the much higher prevalence of orphans in urban areas with high HIV prevalence [33] suggested that HIV/AIDS was likely one the key drivers of parental mortality.

**Table 1:** **Descriptive characteristics [mean (SD) or *n *(Proportion)]**

**Characteristics**	**Full sample**		**Rural**	
	**(N = 1 410)**		**(N = 749)**	
*Child’s demographics*				
Male, n (%)	674	(47.8)	365	(48.7)
Age in months, mean (SD)	74.6	(4.11)	75.3	(4.27)
Father dead, n (%)	99	(7.02)	42	(5.61)
Mother dead, n (%)	27	(1.91)	10	(1.34)
Father and mother dead, n (%)	5	(0.35)	1	(0.13)
*Household-level variables, mean (SD)*				
Highest educational attainment (years)	8.49	(3.62)	7.70	(3.62)
Household size	5.46	(1.93)	5.60	(1.91)
Wealth quintile	2.85	(1.41)	2.23	(1.25)
Number of younger siblings in household	0.68	(0.81)	0.70	(0.84)
Number of older siblings in household	2.00	(1.52)	2.07	(1.51)
*Cluster-level variables, mean (SD)*				
Parasitemia (fraction of MIS 2006 slides positive)	0.14	(0.20)	0.22	(0.22)
Fraction of parents deceased	0.10	(0.09)	0.07	(0.07)
Average educational attainment	8.49	(2.33)	7.70	(2.25)
Average wealth quintile	2.85	(1.04)	2.23	(0.62)
*Outcomes*				
Peabody Picture Vocabulary Test (z-score), mean (SD)	−0.01	(1.01)	−0.04	(1.15)
Task Orientation (z-score), mean (SD)	0.00	(1.00)	−0.18	(1.06)
Tactile Pattern Recognition (z-score), mean (SD)	0.03	(1.00)	0.06	(1.03)
Socio-emotional Development (principal component z-score), mean (SD)	−0.01	(1.00)	−0.03	(1.02)
Fine Motor Skills (z-score), mean (SD)	−0.04	(1.01)	−0.20	(1.07)
Weight in kilograms, mean (SD)	21.58	(9.06)	22.45	(11.52)
Height in centimeters, mean (SD)	117.06	(8.12)	116.70	(7.14)
Stunted, n (%)	160	(0.17)	88	(0.17)
Underweight, n (%)	116	(0.12)	54	(0.10)
Body Mass Index, mean (SD)	15.12	(3.11)	15.36	(3.83)

Adult educational attainment was low, with an average of 8.5% years of schooling completed by the most educated household member. The bottom section of Table 1 summarizes the developmental measures analysed. All perceived and assessed measures of child development were normalized relative to the average of the full ZECDP sample.

The average height of children in the sample was 117 centimetres; the average weight was 21.6 kilograms. Seventeen percent of children in the full sample were stunted (height < = −2SD below reference median), and 12% of children were classified as underweight (weight < = −2SD below reference median). Overall, only minor differences were found with respect to height and weight across children living in rural, and children living in urban areas.

Table 2 shows the associations between malaria exposure and anthropometric outcomes estimated in the multi-level models. All specifications include random effects at the cluster level, district-fixed effects, child sex, child age in months, orphanhood status, highest education in household, household size, household wealth quintile, number of children younger than six years in the household, number of children between six and 18 years old in the household, highest level of education completed by adults in cluster, a proxy for cluster-level mortality of adults and cluster-level wealth quintile. On average, a positive but not statistically significant association was found between early childhood exposure to malaria and delayed physical development. Given that malaria parasitaemia was defined as the fraction of positive malaria tests in the cluster, the reported point estimates imply that children in clusters with positivity rates of 0.72 were on average about 2 cm shorter and 0.4 kg lighter than children growing up in areas without any parasites.

**Table 2:** Adjusted associations between cluster-level parasitaemia and child anthropometrics

	**Weight**^**a**^	**Height**^**a**^	**Underweight**^**b**^	**Stunted**^**b**^
	**(1)**	**(2)**	**(3)**	**(4)**
Full Sample				
Malaria exposure	−0.562	−3.374	2.296	3.000
	(−5.974 - 4.850)	(−11.35 - 4.606)	(0.308 - 17.10)	(0.584 - 15.41)
p-value	0.839	0.407	0.417	0.417
Total *N*	978	968	978	978
Rural Sample				
Malaria exposure	−0.740	−3.110	13.46	4.975
	(−11.04 - 9.564)	(−8.901 - 2.681)	(0.243 - 745.3)	(0.307 - 80.68)
p-value	0.888	0.293	0.204	0.204
Total *N*	533	525	533	533

^a^Columns (1) and (2) display coefficients of linear regression with 95% confidence intervals in parentheses. These specifications include random effects at the cluster level and district fixed effects.

^b^Columns (3) and (4) display Odd Ratios from conditional logit models with district fixed effects and cluster level random effects. All specifications include child sex, age in months, orphanhood status, highest education (years of schooling completed) in household, household size, household wealth quintile, number of children younger than six years old in the household, number of children between six and 18 years old in the household, average highest level of education completed by adults in cluster, a cluster-level parental mortality, and cluster-level wealth quintile average.

As shown in columns (3) and (4) of Table 2, the main differences in these outcomes appeared to come from the left-hand tail of the distribution: keeping all else equal, children growing up in areas with with the highest positivity rates (0.72) had on average 1.6 times the odds of being wasted, and 2.1 times odds of being stunted compared to children with zero positivity rates. While these estimates were large, the null hypothesis of no association could not be rejected. When the sample was restricted to children in rural areas (bottom panel of Table 2), the results did not change qualitatively. Even though the coefficients changed in magnitude, the null hypothesis of equal coefficients could not be rejected due to the rather large confidence intervals generated in the smaller sample.

Table 3 shows the results for the broader set of developmental measures. Column (1) shows the result for the interviewer’s overall assessment of the child’s ability to complete basic tasks (task orientation); column (2) shows the results for the child’s overall socio-emotional development as assessed by the parent or caretaker. The effects on both domains were large: on average, children from areas with 0.72 positivity rates scored 0.85 standard deviations below children from non-malaria areas. Similar differences were found for socio-emotional development. A point estimate of −1.3 implies that children from the most exposed areas (0.72 positivity rate) on average performed 0.91 standard deviations below non-exposed children. Columns (3) and (4) show the associations between malaria and receptive language and fine-motor skills. While both measures displayed negative associations with malaria exposure, the estimated magnitudes were smaller, and the null of zero impact could not be rejected. The results for task orientation and socio-emotional development looked similar for the rural sample, with a slightly smaller coefficient estimated for task orientation, and a larger coefficient estimated for socio-emotional development. The results on the two other developmental outcomes were more mixed: while the estimates suggested a larger (more negative) effect of malaria on fine motor skills in rural areas, the association between receptive language and malaria appeared positive and highly statistically significant in the rural subsample.

**Table 3:** Adjusted associations of cluster-level parasitaemia and child development

	**Task Orientation**	**Socio-emotional Development**	**Receptive Language**	**Fine Motor Skills**
	**(1)**	**(2)**	**(3)**	**(4)**
Full Sample				
Malaria exposure	−1.178	−1.265	−0.127	−0.288
	(−2.323 - -0.0332)	(−2.833 - 0.302)	(−1.291 - 1.037)	(−1.089 - 0.513)
P-value	0.0437	0.114	0.831	0.481
Sample size	1,378	1,396	1,410	1,410
Rural Sample				
Malaria exposure	−0.808	−2.739	1.430	−0.532
	(−1.437 - -0.179)	(−3.296 - -2.181)	(0.780 - 2.080)	(−1.209 - 0.145)
p-value	0.012	0.000	0.000	0.124
Sample size	729	741	749	749

Coefficients of linear random effects regression with 95% confidence intervals in parentheses.

### Relative magnitude of associations

Table 4 compares the estimated malaria impact to the relative impact of household wealth and education. The first row of Table 4 shows the developmental change predicted by moving a child from the area with the highest parasitaemia (positivity rate 0.72) to an area with zero positivity. The second row shows the predicted impact from increasing adult education from zero (which is still very common in Zambia) to seven years of schooling (which corresponds to completed primary schooling). The third row of Table 4 shows the predicted improvements with an increase in household wealth. More specifically, the numbers displayed reflect the marginal increase in child development predicted by moving a child from the poorest quintile to the richest. As Table 4 shows, the predicted impact of additional education was found to be positive across all domains, with an average impact of less than a quarter of a standard deviation on the four non-anthropometric domains. In the rural sample, education appeared to have a larger positive effect on anthropometrics, with gains comparable in magnitude to the gains attainable through reductions in malaria exposure.

**Table 4:** Relative magnitude of estimates

	**Weight**	**Height**	**Task Orientation**	**Socio-emotional**	**Receptive Language**	**Fine Motor Skills**
	**(1)**	**(2)**	**(3)**	**(4)**	**(5)**	**(6)**
Full Sample						
Eliminating malaria^a^	0.40	2.43	0.85	0.91	0.09	−0.21
	(4.301 -3.492)	(8.172 -3.316)	(1.673 -0.024)	(2.04 -0.217)	(0.93 -0.747)	(−0.784 0.369)
Increasing education^b^	0.42	1.13	0.24	0.09	0.23	0.23
	(−1.309 0.98)	(−1.484 0.543)	(0.118 0.358)	(−0.007 0.2)	(0.116 0.351)	(0.098 0.354)
Increasing wealth^c^	2.10	−0.19	0.31	0.38	0.12	0.39
	(−0.361 4.569)	(−2.276 1.903)	(0.108 0.508)	(0.204 0.565)	(−0.07 0.315)	(0.177 0.597)
Rural Sample						
Eliminating malaria^a^	0.53	2.24	0.58	1.97	−1.03	−0.38
	(7.949 -6.886)	(6.409 -1.93)	(1.035 0.129)	(2.373 1.57)	(−0.562 -1.498)	(−0.87 0.104)
Increasing education^b^	0.60	2.61	0.37	0.11	0.31	0.34
	(−2.17 3.367)	(1.05 4.179)	(0.191 0.554)	(−0.049 0.267)	(0.128 0.495)	(0.149 0.531)
Increasing wealth^c^	0.31	0.38	0.32	0.47	0.11	0.27
	(−1.247 6.701)	(−2.62 1.941)	(0.031 0.613)	(0.212 0.726)	(−0.168 0.392)	(−0.02 0.564)

^a^The numbers reported in this row correspond to the co-efficients reported in Tables 2 and 3 multiplied by −0.72, the highest level of malaria parasitaemia the sample.

^b^The numbers reported in this row correspond to coefficients estimated for highest education in the household from the specifications reported in Tables 2 and 3 multiplied by 7, equivalent to increasing education from 0 to completed primary schooling.

^c^The numbers reported in this row correspond to the estimated impact achievable by moving a child from a household in the poorest wealth quintile to a household in the top quintile as estimated in the empirical models underlying Tables 2 and 3.

95% Confidence intervals in parenthesis.

The effects of wealth were more mixed. On average, wealth was weakly associated with weight (*β* = 2.1, p-value 0.094), but did not show any statistically significant association with height. Accordingly, increases in household wealth were predicted to positively contribute to children’s weight, but not to children’s height. Wealth appeared to have a mostly positive effect on other domains of development however, with shifts in household wealth predicted to improve child development between 0.12 (receptive language) and 0.39 (fine motor skills) standard deviations. Overall, the basic comparisons reported in Table 4 suggest that the average improvements in child development achievable through reductions in malaria exposure are comparable in magnitude or larger than the improvements achievable through education and the improvements achievable through higher household income or wealth.

## Discussion

The results presented in this paper demonstrate associations between early childhood exposure to malaria and some, but not all domains of children’s pre-school development. Generally, the estimated associations with malaria appear large compared to the associations with education and wealth, the two covariates generally considered to be the principal determinants of child development. Even though the results appear plausible, it appears important to highlight a few issues affecting the interpretation of the results. First, while the malaria exposure measure used in the empirical analysis is likely an accurate assessment of the cluster-level exposure to malaria, it does not necessarily measure the actual exposure of each child. To the extent that some parents or guardians managed to shield their children from the parasite, the analyses presented here do not estimate the effect of exposure to malaria in an uncontrolled environment, but, rather, the effect of malaria exposure conditional on the average parental efforts to protect their children. The effect of malaria on child development may also be mediated by attendance of early childhood programmes, an institution less than 30% of Zambian children currently benefit from [34].

A second, and related point, regards the duration of malaria exposure. Given the large national efforts against malaria, the 2006 prevalence rate is likely a decent proxy for malaria exposure in the first or first two years of these children’s life, but not necessarily a good measure of malaria exposure during early childhood more generally. If continued exposure to malaria matters beyond age two for child development, the results may underestimate the true effect of malaria to the extent the national campaign succeeded in lowering the prevalence of the disease.

Third, while the 2006 parasitaemia data are used as proxy for the child’s exposure to malaria, it appears likely that malaria prevalence during that period is correlated with malaria exposure in pregnancy, so that some of the effects identified might reflect malaria exposure in pregnancy, rather than the effects of the child’s exposure to the parasite.

Fourth, even though a large set of controls is included in the main specification to reduce the risk of confounding, the cross-sectional nature of this study does not rule out residual correlations between unobservable household or cluster characteristics and the malaria measure. If these unobservable factors have a causal effect on child outcomes, the effects of malaria may be over- or underestimated even in the fully specified model.

Last, by focusing on children surviving up to age five, the study does not only abstract from the important mortality impact malaria has in the country, but also identifies the marginal impact of malaria among the selected sample of children surviving initial exposure to the disease. To the extent that these surviving children are more resilient to the parasite than the general population of children, the study may underestimate the true effect of malaria on child development. From a broader policy perspective, the empirical strategy employed in this paper was clearly not designed to fully measure the burden of malaria. Rather, this study illustrates the burden of malaria for child development, an aspect which should be considered for the broader health and economic costs generated by the disease in order to generate a comprehensive estimate of the true societal cost of malaria.

## Conclusion

Millions of children continue to be exposed to malaria in Africa today. The results of this study suggest that exposure to the disease is not only a threat to children’s survival as extensively documented in the literature, but may also undermine their cognitive development.

## Competing interests

The authors declare no competing interests.

## Authors’ contributions

GF conceived of the study, supervised its design and implementation, and drafted the manuscript. AO took the lead in the empirical analysis of the data and literature review. MH and JM coordinated the MIS data collection, blood testing and merging of the MIS with ZECDP data set. JM provided the sampling frame for the ZECDP as well as all spatial information used in the analytical part. BM supervised all aspects of ZECDP data collection. All authors reviewed and approved the final manuscript.
